# Exposure to Chinese Famine during Early Life Increases the Risk of Fracture during Adulthood

**DOI:** 10.3390/nu14051060

**Published:** 2022-03-03

**Authors:** Zumin Shi, Xinyu Shi, Alice F. Yan

**Affiliations:** 1 Human Nutrition Department, College of Health Sciences, QU Health, Qatar University, Doha 2713, Qatar; 2 Transformation & Operation, Deloitte, Canberra, ACT 2609, Australia; xinyu.shi@quantium.com.au; 3 Department of Medicine, Division of Internal Medicine, Center for Advancing Population Science, Medical College of Wisconsin, Milwaukee, WI 53226, USA; aliceyan@mcw.edu

**Keywords:** famine, fracture, adults, cohort study, Chinese

## Abstract

This study focused on identifying whether exposure to the Chinese Great Famine (1959–1961) in early life amplified the potential for fractures in adulthood. The survey was conducted using data from the 1997–2015 China Health and Nutrition Survey (CHNS)—5235 adults born between 1954 and 1964 were selected as the sample size. Fracture was defined based on self-report. Those born from 1962–1964 were treated as non-exposure group. Those with exposure to famine were divided into four subgroups: Fetal, early childhood, mid-childhood, and late childhood cohorts. The association between the groups and fracture was determined using Cox regression. In follow-up data (mean of 11 years), fractures were identified in 418 of the participants. The incidence of fracture was 8.7 in late childhood, 8.1 in mid-childhood, 8.3 in early childhood, 7.0 in fetal, and 5.4 in non-exposed cohorts per 1000 person-year. Compared with the non-exposed group, the famine-exposed groups had an increased risk of developing fracture in adulthood with hazard ratio (HR) and 95% CI of 1.29 (0.90–1.85), 1.48 (1.08–2.03), 1.45 (1.02–2.06), and 1.54 (1.08–2.20), respectively. The positive link of famine exposure to risk of fracture occurred primarily in those participants with a modern diet who lived in urban areas. In conclusion, the risk of fracture in Chinese adults is associated with famine exposure.

## 1. Introduction

Fracture is a major contributor to pain, decreased function, reduced quality of life, and elevated risk of mortality. Based on the Chinese National Survey (CNS), the incidence rate of traumatic fractures in the appendages and trunk was 3.21 per 1000 people in 2014 [1]. The majority of traumatic fractures are due to slip, trip or fall (57.7%). China used to be a country with a low risk of fracture. In recent decades, the burden of fracture increased in mainland China, but with a large geographic variation [2,3]. Urbanization, motorization, and nutrition transition are thought to be responsible for the change [3].

Meanwhile, data from epidemiological studies (e.g., famine studies) and animal models indicate that early life malnutrition is a significant indicator of health in adulthood [4,5,6,7]. China experienced a 3-year famine between 1959 and 1961. It is the longest famine ever recorded in human history. In addition, it led to approximately 30 million excess deaths [8,9]. Since 2012, there have been a growing number of research studies on the effects of exposure to the Chinese famine (1959–1961) in early life exposure. Findings from these studies indicate hypothetically increased risks of hypertension [10], diabetes [11,12,13,14], overweight/obesity [15], as well as metabolic syndrome [16,17] in adulthood. Moreover, it has been shown that famine exposure exacerbated the association between conventional chronic disease risk factors and chronic diseases [18,19]. Case studies from different countries showed that in utero famine exposure increased the risk of adult osteoporosis and fracture [20]. Two studies in China examined the long-term effects of early famine experience and the risk of osteoporosis [21,22] and supported the link. Furthermore, results from the Kailuan study [23] and Kadoorie Biobank (CKB) cohort study in China [24] suggested that in utero and early childhood famine exposure increased the risk of rheumatoid arthritis (RA) in adulthood. Osteoporosis is known to increase the risk of fracture [25]. However, it is unknown whether famine exposure increases the risk of fracture in Chinese adults.

This study aimed to (1) evaluate the link between risk of fracture in adulthood and exposure to famine in early life; (2) test if the hypothesized association is modified by lifestyle among Chinese adults participating in the China Health and Nutrition Survey (CHNS).

## 2. Materials and Methods

### 2.1. Study Design and Sample

**Figure 1:**
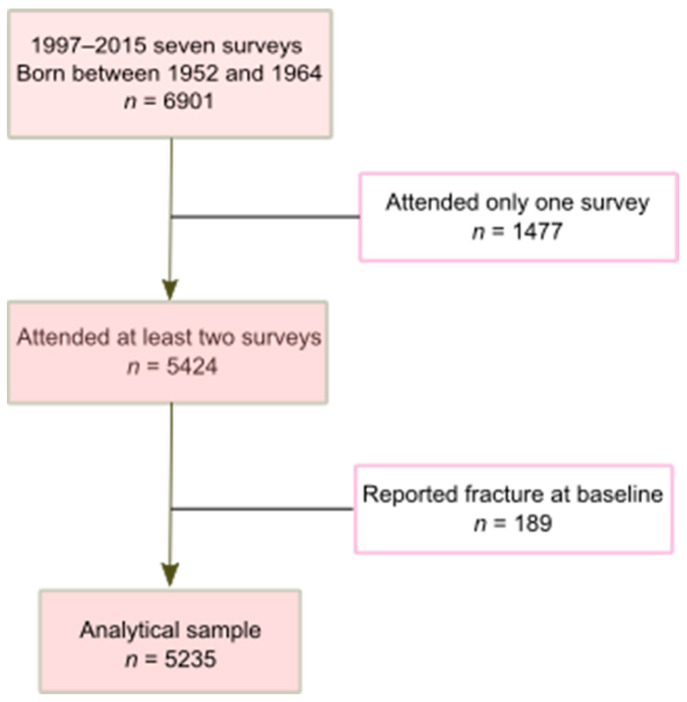
Sample selection process (CHNS 1997–2015).

This study used data from the nationwide China Health and Nutrition Survey (CHNS), which contained 18-years of follow-up data (1997, 2000, 2004, 2006, 2009, 2011, and 2015) [26,27,28]. As a prospective cohort study, the CHNS uses a multistage random-cluster sampling process to select households in both rural and urban areas [29]. As a result, the sample represents a set of large provinces and municipal cities with a range of geography (i.e., 12 provinces in the current analysis), economic development, and demographic variation in China [27]. Ten waves of data were collected between 1989 and 2015. Participants had the option of joining or leaving the study during any of the survey waves. Of the 6901 participants born between 1952 and 1964, 1666 were excluded in the analysis as 1477 had completed the survey only once and 189 reported fracture during the first survey since 1997 (Figure 1). In total, the current analysis included 5235 participants. As the data for this study were publicly available and de-identified, the study presented here was exempt from institutional review board oversight.

### 2.2. Outcome Variable: Fracture

The fracture was measured by self-report in each wave using the following question: “Have you ever had a fracture?” along with gathering data on the age at which the initial fracture occurred [30]. The occurrence of fracture was demarcated if a participant informed the study interviewers of a new history of fracture.

### 2.3. Exposure Variable: Great Chinese Famine

The Great Famine in China lasted from 1959 to 1961. This 3-year period was used to identify the sample population. Birth year was used to define exposure to the famine in early life. In line with prior studies concentrating on exposure to the Great Famine in China [16,31,32], subjects were divided into five famine exposure subgroups according to their birthdates and corresponding exposure period: (1) Adults born between 1962 and 1964 were classified as non-exposure group; (2) adults born between 1959 and 1961 were classified as fetal exposure group. The assumption was that the participants’ mothers would have been impacted by the famine all throughout the participants’ gestation. Then, those subjects born between 1952 and 1958 were divided into 2-year groups [16] and were classified based on the level of childhood exposure: (3) Early childhood (born from 1956 to 1958, exposed to famine at ages 1–3), (4) mid-childhood (born from 1954 to 1956, exposed to famine at ages 3–5), and (5) late childhood (born from 1952 to 1954, exposed to famine at ages 5–7).

The famine used in this study has also been referred to as the Great Leap Forward Famine (1959 and 1961) [33]. Although the Chinese famine impacted all of mainland China, the severity differed across different provinces [9]. The excess death rate for each province was utilized as a proxy for determining the severity of the famine [16,23,32]. The excess mortality (50%) rate indicated a threshold for distinguishing which regions were severely affected or less affected [12,23]. Based on the literature [9], we presented the excess mortality in each participating province in Figure S1.

#### Measures of Dietary Patterns and Nutrient Intake

Individual dietary intake was determined using a 24-h dietary recall method and collected over 3 days at each wave. This was also supported using dietary records that were maintained by individuals with the final dietary data, including the type and amount of food, the type of meal, and the place of consumption. Condiment consumption (e.g., cooking oil) for every member of the household was projected through a weighed calculation of household intake (estimating the energy intake of individual) [34]. In addition, daily household food consumption was established by calculating updates (using the Chinese Food Composition Table) to the home food inventory.

In the dietary pattern analysis, food intake was divided initially into 35 groups based on nutrient profile or culinary use, and the average food intake for individuals (gram/day) was calculated for each wave. Across the six waves (1997–2011), dietary patterns were identified using a factor analysis with a standard principal component analysis using an orthogonal (varimax) rotation. Details for the factor analysis were reported elsewhere [30]. There were two dietary patterns acknowledged: Pattern 1, which is referred to as “traditional south”, heavily features pork, rice, and vegetables along with a reduced consumption of wheat. Pattern 2, which is referred to as the “modern dietary” pattern, includes high intake of milk/soy milk, fast food, eggs, fruit, fried foods, beer, and different types of meat. Factor loadings of the dietary patterns in the current analytical sample were presented in Table S1.

### 2.4. Covariates

Detailed data on sociodemographic characteristics, lifestyle variables, physical measurements, and chronic conditions were collected at each wave. Data on participants were divided into tertiles for income (low, middle, and high). Then, academic attainment was divided into three levels: Low (illiterate or primary school), medium (junior middle school), and high (high middle school or higher). Lifestyle variables, included smoking, alcohol consumption, and physical activity levels. The level and duration of physical activity was assessed using self-reports on four major types of activities (occupational, domestic, transportation, and leisure time physical activity) [35]. The residency status of participants was classified by dividing the group into two categories (urban and rural).

Height and weight measurements were carried out following a protocol endorsed by the World Health Organization (WHO). Body mass index (BMI) was calculated as weight (kg) divided by the square of height (m). Overweight was defined as BMI ≥ 24 kg/m^2^.

### 2.5. Statistical Analysis

To compare differences between categorical variables, the Chi-square test was used, while analysis of variance (ANOVA) was used for continuous variables. Multivariable Cox regression was used to examine the association between famine exposure and fracture, adjusting for covariates. Herein, three models were used in the analysis: Model 1 was adjusted for gender; model 2 was adjusted for a number of different traits, including alcohol consumption, smoking patterns, educational background, urban/rural location, and level of physical activity; and finally, model 3 was adjusted for dietary patterns. In sensitivity analysis, we excluded those below the age of 35 years. Cox proportional hazard assumption was investigated by a visual inspection of log–log plots generated by stphplot syntax in STATA 17 (Stata Corporation, College Station, TX, USA).

We tested the multiplicative interaction between dietary pattern, residence, gender, overweight, area level famine severity, and famine exposure in relation to fracture risk by including a cross-product term in the main multivariable model (model 3). Missing value was not a problem in the study as a face-to-face interview method was used. Therefore, multiple imputation was not conducted in the analysis. In a parsimonious model attuned for characteristics, such as gender, education, smoking history, urban/rural location, income, energy intake, and famine severity, a subgroup analysis was conducted to examine the link between high intake of a modern dietary pattern (more than medium score) and risk of fracture among famine exposure cohorts. The results were visually presented in a forest plot.

The analyses were accomplished with the use of STATA 17 (Stata Corporation, College Station, TX, USA) and R Statistical Software (version 4.1.0; R Foundation for Statistical Computing, Vienna, Austria).

## 3. Results

### Sample Description

Of the 5235 participants, 840 (16.1%) had famine exposure during the fetal stage. Notably, there were no significant differences at baseline, such as in residence, smoking, energy or macronutrient intake across different famine exposure groups (Table 1). In a cross-group comparison, there was a higher level of educational attainment for the fetal exposure group. At the same time, the fetal exposure group showed the greatest prevalence of overweight/obesity among exposure groups.

**Figure 2:**
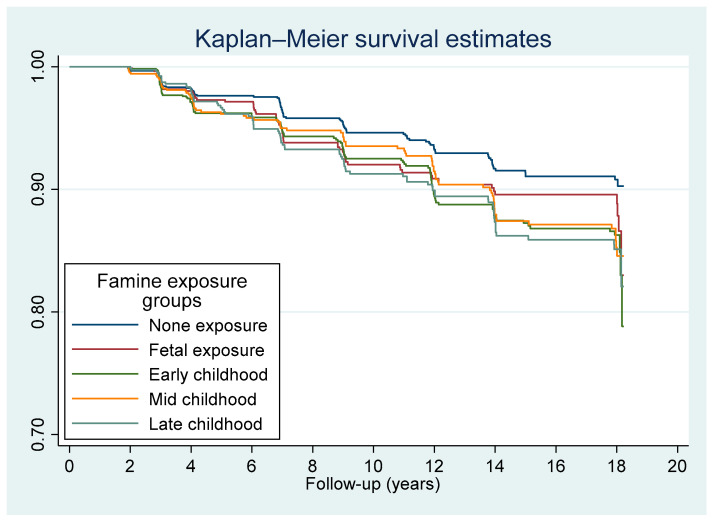
Kaplan–Meier survival curve of fracture by famine exposure among the participants that attended CHNS 1997–2015 (*n* = 5235).

A total of 418 participants developed fracture during the 57,468 person-year follow-up. The average follow-up occurred 11.0 (SD 5.9) years after the initial study data were gathered. The mean age was 49.4 (SD 6.3) years when the fracture occurred during follow-up. The incident rate of fracture varied by region (Figure S2). Famine exposure was positively associated with incident fracture (Figure 2). The incidence of fracture in adulthood for those in the late childhood, mid-childhood, early childhood, fetal, and non-exposed cohorts was 8.7, 8.1, 8.3, 7.0, and 5.4 per 1000 person-year, respectively (Table 2). In contrast with the non-exposed group, the famine-exposed group demonstrated an elevated risk of developing fracture with a hazard ratio (HR) and 95% CI of 1.29 (0.90–1.85), 1.48 (1.08–2.03), 1.45 (1.02–2.06), and 1.54 (1.08–2.20) among adults in fetal, early childhood, mid-childhood, and late childhood cohorts, respectively. In a sensitivity analysis, including those age ≥ 35 years, the HR (95% CI) for fracture was 1.47 (1.01–2.15), 1.71 (1.22–2.40), 1.68 (1.16–2.43), and 1.79 (1.23–2.60) for participants exposed to the famine during fetal, early childhood, mid-childhood, and late childhood, respectively.

**Figure 3:**
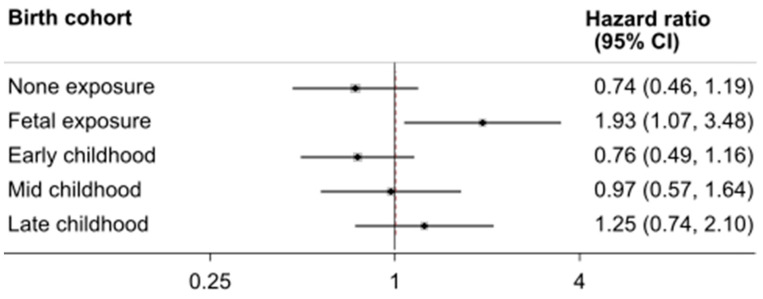
Subgroup analyses of the association between high intake of modern dietary pattern and fracture by famine exposure cohorts. Model was adjusted for gender, smoking, education, urban/rural, income, energy intake, and area level famine severity; *p* for interaction 0.043.

A significant interaction between famine exposure and location of residence was observed in relation to fracture risk (Table 3). The link between early life exposure to famine and fracture risk was only observed among urban residents. In fact, all of the famine exposure groups had a nearly doubled risk of fractures in comparison with the non-exposure group. A similar interaction was observed between exposure to famine and modern dietary pattern. The association between famine exposure and fracture was found among those with high intake of the modern diet, but not those with a more traditional dietary pattern. Furthermore, famine modified the links between dietary pattern and risk of fracture (Figure 3). The only observed association between dietary pattern and fracture risk was in the fetal exposure group.

Famine severity was positively associated with fracture with an HR of 1.53 (95% CI 1.04–2.23) after adjusting for sociodemographic characteristics, lifestyle factors, BMI, and dietary patterns (data not shown). However, there was no significant interaction between famine severity and famine exposure in relation to incident fracture.

There was no significant interaction between genders, overweight/obesity, traditional dietary pattern, and famine exposure.

## 4. Discussion

In this cohort study, we found that early life exposure to famine (1959–1961) in China was positively linked with the risk of fracture irrespective of BMI and elements of personal lifestyle. There was an interaction between famine exposure with residence and modern dietary pattern in relation to fracture. The positive association between early life famine exposure and adulthood fracture risk was only observed in the urban area and those who followed a modern dietary pattern.

### 4.1. Comparison with Other Studies

This paper presents a first-of-its-kind study that successfully identifies a link between fracture among Chinese adults and early life famine exposure. The findings are supported by prior famine studies, as well as fetal origins of adult disease (FOAD) [4,5,6,7]. Case studies from different countries show that early famine or starvation affects bone health. For example, in one study, Holocaust survivors (age ~ 70 years) were more likely to have osteoporosis than the control (54.8% vs. 39.7%) [36]. A study conducted in 1826 on Hong Kong women aged ≥ 65 years found that self-reported famine history (defined as caloric restriction for at least 1 year during World War II) decreased bone mineral density (BMD) and increased the risk of osteoporosis [22]. Another study conducted in Fujian, China, found that early life exposure to Chinese famine was associated with poor bone health [21]. Furthermore, the gender difference in the association between osteoporosis and BMD was found in the Fujian study [21]. The association was only significant in women, but not in men. In women, those born during the famine were 3.67 (95% CI 1.23–10.96) times more likely to have osteoporosis as compared with the non-exposed group [21]. Nevertheless, this study uses a sample that covers only one province that had a follow-up at 3 years. The Fujian study showed a positive association between famine exposure and possible vertebral fracture (as indicated by a 2 cm reduction of height in 3 years of follow-up).

### 4.2. Potential Mechanisms of Early Life Famine Exposure Related Adult Fracture

There are a number of mechanisms that could potentially explain the link occurring between fracture risk in adults and early life famine exposure. First, human famine studies as well as animal models suggest that there are common mechanisms linking nutrition in early life and age-associated diseases, e.g., tissue structure, epigenetic regulation, and accelerated cellular aging [37]. A series of clinical studies in different countries support the link between the development of osteoporosis and maternal nutrition [38]. Second, nutrition in early life determines the quality and quantity of bone in later life. For example, during intrauterine and early postnatal life, human skeletal growth is programmed [39]. Skeletal size in adulthood is associated with growth in infancy. Maternal vitamin D deficiency leads to a reduced bone mass in the offspring [40]. Calcium intake in early life is a key determinant of bone mineral density [41]. Third, early life malnutrition may cause gut microbiota immaturity [42]. Changes to gut microbiota in older adults are also linked with chronic diseases [43] and bone mineral density (BMD) [44]. Finally, famine increases the risk of NCDs, including diabetes [14], rheumatoid arthritis [23], and hypertension [10], which are risk factors for fracture.

The interaction between famine exposure and modern dietary pattern is intriguing. It aligns with a prior study that found early life famine exposure in China was linked with an increased risk of diabetes, particularly in adulthood for those who habitually consume a modern diet [12]. In the CHNS, there was a positive association between fracture risk and modern diet consumption in adulthood [30].

The interaction between famine exposure and residence is biologically plausible as people living in the urban area have higher risk of fracture due to various reasons, including limited space for physical activity, more access to unhealthy diet, and increased stress. Mismatch of the living environment at different life stages is an important risk factor of many chronic diseases [45]. Although there was no statistically significant interaction between famine exposure and gender, the association between famine exposure and fracture was only significant in women, but not in men. This is supported by the fact that women had higher risk of fracture than men.

### 4.3. Limitations and Strengths

This study has a number of strengths, including (1) a geographically diverse sample with a long follow-up; (2) NCDs, sociodemographic traits, and lifestyle characteristics are readily available through the CHNS database; (3) dietary intake was measured by repeated 3-day food recall in a combination of household food inventories. This study’s primary constraint is that the data on fractures provide no further information on the type of fracture. Under-reporting of fracture may be more common among rural area than urban area due to the difference in the access to medical service. However, recall bias of the outcome variable is less likely, since fracture is a major life event. We do not have information on the famine severity that each individual experienced. The use of area famine severity cannot reflect the individual degree of malnutrition during early life. A relatively small sample of participants was identified as stemming from areas with less severe famines. Although the study included 12 provinces in China, it did not recruit participants in the western part of China. The results may not be generalizable. Finally, recall bias may exist in the 3-day food intake measurement. However, the total food intake during the 3 days in the household was measured by the weighing method and reliable.

## 5. Conclusions

In this study of participants in the China Health and Nutrition Survey, a strong association was found between early life famine exposure and fracture risk in adulthood among urban residents and those with a modern dietary pattern. Early life nutrition should be considered in the prevention of fracture in adults.

## Figures and Tables

**Table 1:** Baseline sample characteristics by early life famine exposure: China Health and Nutrition Survey (*n* = 5235).

Factor	None Exposure (*n* = 1505)	Fetal Exposure (*n* = 840)	Early Childhood (*n* = 1204)	Mid-Childhood (*n* = 866)	Late Childhood (*n* = 820)	*p*-Value ^1^
Birth year	1962–1964	1959–1961	1956–1958	1954–1955	1952–1953	
Age (years), mean (SD)	37.7 (5.4)	41.2 (5.7)	44.3 (5.8)	46.7 (5.9)	48.7 (5.9)	<0.001
Overweight	24.7%	30.2%	28.9%	26.3%	28.9%	0.043
BMI (kg/m^2^), mean (SD)	23.2 (3.1)	23.5 (3.3)	23.4 (3.2)	23.2 (3.1)	23.5 (3.1)	0.093
Height (cm), mean (SD)	162.5 (7.9)	161.9 (8.0)	161.3 (8.1)	161.0 (8.1)	160.7 (8.3)	<0.001
Weight (kg), mean (SD)	61.5 (10.7)	61.8 (10.9)	61.1 (10.3)	60.3 (10.2)	60.9 (10.0)	0.043
Women	51.2%	51.0%	51.3%	49.8%	52.0%	0.927
Income						0.594
Low	26.9%	24.7%	27.2%	26.9%	26.3%	
Medium	34.6%	35.6%	32.9%	32.8%	31.4%	
High	38.5%	39.7%	39.9%	40.3%	42.4%	
Education						<0.001
Low	19.4%	23.0%	32.7%	44.0%	51.7%	
Medium	42.2%	32.0%	35.0%	31.2%	30.6%	
High	38.4%	45.0%	32.3%	24.8%	17.7%	
Rural residence	59.3%	59.4%	58.2%	58.0%	61.4%	0.625
Severe famine area	85.0%	79.3%	80.7%	80.8%	80.9%	0.003
Smoking						0.161
Non smoker	66.4%	63.8%	65.4%	65.0%	62.7%	
Ex-smokers	0.8%	0.9%	1.6%	1.7%	2.1%	
Current smokers	32.8%	35.3%	33.0%	33.3%	35.1%	
Physical activity (MET-hours/week), mean (SD)	149.3 (112.0)	157.1 (117.0)	144.0 (109.5)	149.6 (116.1)	141.7 (118.6)	0.063
Energy intake (kcal/d), mean (SD)	2235.1 (756.0)	2241.4 (692.1)	2241.6 (1062.4)	2213.5 (734.7)	2218.2 (705.4)	0.923
Fat intake (g/d), mean (SD)	68.9 (36.9)	71.0 (35.5)	72.8 (100.3)	70.6 (52.9)	68.4 (35.5)	0.481
Protein intake (g/d), mean (SD)	70.3 (43.8)	71.3 (22.6)	70.1 (23.6)	68.3 (23.7)	69.6 (24.8)	0.375
Carbohydrate intake (g/d), mean (SD)	328.4 (135.9)	324.4 (132.1)	319.8 (127.2)	320.6 (126.5)	325.3 (133.0)	0.520
Calcium intake (mg/d), mean (SD)	372.8 (339.6)	381.3 (205.3)	392.0 (228.8)	376.8 (215.4)	405.1 (349.8)	0.090
Traditional south dietary pattern, mean (SD)	0.0 (1.0)	−0.0 (0.9)	0.0 (1.0)	0.0 (1.0)	0.1 (1.0)	0.463
Modern dietary pattern, mean (SD)	−0.1 (0.9)	0.0 (1.1)	−0.0 (1.1)	−0.1 (1.1)	−0.1 (1.0)	0.037
Survey year						<0.001
1997	54.2%	52.1%	53.4%	55.5%	55.2%	
2000	14.0%	14.0%	11.9%	11.3%	10.6%	
2004	6.6%	3.9%	5.5%	5.1%	5.5%	
2006	4.9%	5.8%	3.3%	2.4%	2.1%	
2009	6.7%	7.7%	7.1%	5.0%	5.1%	
2011	13.6%	16.3%	18.9%	20.7%	21.5%	

^1^ *p* from ANOVA for continuous measures or Chi-square tests for categorical data.

**Table 2:** Hazard ratio (95% CI) for fracture by early life Chinese famine (1959–1961) exposure.

	None Exposure (*n* = 1505)	Fetal Exposure (*n* = 840)	Early Childhood (*n* = 1204)	Mid-Childhood (*n* = 866)	Late Childhood (*n* = 820)
No. of cases	91	64	108	78	77
Person-years	16,858	9111	12,976	9640	8884
Incidence rate (per 1000 person-year)	5.4	7.0	8.3	8.1	8.7
Model 1 ^1^	1.00	1.31 (0.95–1.81)	1.56 (1.18–2.06)	1.49 (1.10–2.02)	1.61 (1.19–2.18)
Model 2 ^2^	1.00	1.29 (0.90–1.85)	1.48 (1.08–2.03)	1.45 (1.02–2.06)	1.53 (1.07–2.19)
Model 3 ^3^	1.00	1.29 (0.90–1.85)	1.48 (1.08–2.03)	1.45 (1.02–2.06)	1.54 (1.08–2.20)
Model 3 + age ≥ 35 years	1.00	1.47 (1.01–2.15)	1.71 (1.22–2.40)	1.68 (1.16–2.43)	1.79 (1.23–2.60)

^1^ Model 1 was adjusted for gender. ^2^ Model 2 was adjusted for smoking, alcohol drinking, education, urban/rural, and physical activity (MET hours/week). ^3^ Model 3 was adjusted for dietary patterns.

**Table 3:** Hazard ratio (95% CI) for incident fracture by early life famine exposure groups and residence and dietary patterns ^1^.

	None Exposure (*n* = 1505)	Fetal Exposure (*n* = 840)	Early Childhood (*n* = 1204)	Mid-Childhood (*n* = 866)	Late Childhood (*n* = 820)
Residence					
Urban	1.00	2.29 (1.18–4.48)	2.59 (1.40–4.80)	2.8 (1.47–5.31)	2.41 (1.47–5.31)
Rural	1.00	1.06 (0.69–1.64)	1.2 (0.83–1.75)	1.05 (0.68–1.61)	1.24 (0.68–1.61)
*p* for interaction		0.076	0.078	0.026	0.140
Modern dietary pattern					
High modern diet	1.00	1.99 (1.20–3.32)	1.91 (1.18–3.08)	1.96 (1.18–3.28)	1.96 (1.18–3.28)
Low modern diet	1.00	0.88 (0.51–1.50)	1.21 (0.79–1.86)	1.09 (0.67–1.77)	1.26 (0.67–1.77)
*p* for interaction		0.030	0.148	0.059	0.111
Famine severity					
Less severe	1.00	2.77 (0.89–8.65)	2.41 (0.79–7.35)	3.1 (0.99–9.70)	1.14 (0.99–9.70)
Severe	1.00	1.21 (0.83–1.78)	1.43 (1.03–2.00)	1.34 (0.93–1.94)	1.58 (0.93–1.94)
*p* for interaction		0.277	0.518	0.281	0.585
Overweight					
No	1.00	1.54 (1.02–2.35)	1.41 (0.96–2.08)	1.32 (0.86–2.02)	1.69 (0.86–2.02)
Yes	1.00	0.85 (0.41–1.77)	1.64 (0.93–2.90)	1.8 (0.97–3.33)	1.25 (0.97–3.33)
*p* for interaction		0.320	0.451	0.216	0.268
Sex					
Men	1.00	1.14 (0.71–1.85)	1.33 (0.88–2.00)	1.20 (0.75–1.90)	1.30 (0.75–1.90)
Women	1.00	1.57 (0.90–2.73)	1.65 (0.99–2.75)	1.83 (1.07–3.14)	1.86 (1.07–3.14)
*p* for interaction		0.320	0.451	0.216	0.268

^1^ Model was adjusted for gender, smoking, alcohol drinking, education, income, physical activity, BMI, and dietary patterns. Stratification variables were not adjusted in the corresponding models.

## Data Availability

The current research used data from the China Health and Nutrition Survey (CHNS). Data are made publicly and freely available without restriction at https://www.cpc.unc.edu/projects/china (accessed on 5 February 2022).

## Supplementary Materials

The following supporting information can be downloaded at: https://www.mdpi.com/article/10.3390/nu14051060/s1. Figure S1: Study sites of China Health and Nutrition Survey; Figure S2: Incidence rate (per 1000) of fracture by study region among participants born between 1952 and 1964 who attended the China Health and Nutrition Survey between 1997 and 2015; Table S1: Factor loadings of dietary patterns among participants born between 1952 and 1964 who attended the China Health and Nutrition Survey between 1997 and 2015.

## References

[1] Chen W., Lv H., Liu S., Liu B., Zhu Y., Chen X., Yang G., Liu L., Zhang T., Wang H. (2017). National incidence of traumatic fractures in China: —A Retrospective Survey of 512,187 Individuals. Lancet Glob. Health.

[2] Yu F., Xia W. (2019). The epidemiology of osteoporosis, associated fragility fractures, and management gap in China. Arch. Osteoporos..

[3] Gong X.F., Li X.P., Zhang L.X., Center J.R., Bliuc D., Shi Y., Wang H.B., He L., Wu X.B. (2021). Current status and distribution of hip fractures among older adults in China. Osteoporos. Int..

[4] Godfrey K.M., Barker D.J. (2000). Fetal nutrition and adult disease. Am. J. Clin. Nutr..

[5] Gluckman P.D., Hanson M.A., Cooper C., Thornburg K.L. (2008). Effect of In Utero and Early-Life Conditions on Adult Health and Disease. N. Engl. J. Med..

[6] Hales C.N., Barker D.J.P. (2001). The thrifty phenotype hypothesis. Br. Med. Bull..

[7] Roseboom T.J., Painter R.C., van Abeelen A.F., Veenendaal M.V., de Rooij S.R. (2011). Hungry in the womb: —What are the Consequences? Lessons from the Dutch Famine. Maturitas.

[8] Smil V. (1999). China’s great famine: 40 years later. BMJ.

[9] Luo Z., Mu R., Zhang X. (2006). Famine and Overweight in China. Rev. Agric. Econ..

[10] Huang C., Li Z., Wang M., Martorell R. (2010). Early Life Exposure to the 1959–1961 Chinese Famine Has Long-Term Health Consequences. J. Nutr..

[11] Meng R., Lv J., Yu C., Guo Y., Bian Z., Yang L., Chen Y., Zhang H., Chen X., Chen J. (2018). Prenatal famine exposure, adulthood obesity patterns and risk of type 2 diabetes. Int. J. Epidemiol..

[12] Li Y., He Y., Qi L., Jaddoe V.W., Feskens E.J., Yang X., Ma G., Hu F.B. (2010). Exposure to the Chinese Famine in Early Life and the Risk of Hyperglycemia and Type 2 Diabetes in Adulthood. Diabetes.

[13] Wang J., Li Y., Han X., Liu B., Hu H., Wang F., Li X., Yang K., Yuan J., Yao P. (2016). Exposure to the Chinese Famine in Childhood Increases Type 2 Diabetes Risk in Adults. J. Nutr..

[14] Zimmet P., Shi Z., El-Osta A., Ji L. (2020). Chinese Famine and the diabetes mellitus epidemic. Nat. Rev. Endocrinol..

[15] Wang Y., Wang X., Kong Y., Zhang J.H., Zeng Q. (2010). The Great Chinese Famine Leads to Shorter and Overweight Females in Chongqing Chinese Population After 50 Years. Obesity.

[16] Li Y., Jaddoe V.W., Qi L., He Y., Wang D., Lai J., Zhang J., Fu P., Yang X., Hu F.B. (2011). Exposure to the Chinese Famine in Early Life and the Risk of Metabolic Syndrome in Adulthood. Diabetes Care.

[17] Zheng X., Wang Y., Ren W., Luo R., Zhang S., Zhang J.H., Zeng Q. (2012). Risk of metabolic syndrome in adults exposed to the great Chinese famine during the fetal life and early childhood. Eur. J. Clin. Nutr..

[18] Shi Z., Nicholls S.J., Taylor A.W., Magliano D.J., Appleton S., Zimmet P. (2018). Early life exposure to Chinese famine modifies the association between hypertension and cardiovascular disease. J. Hypertens..

[19] Shi Z., Ji L., Ma R.C.W., Zimmet P. (2020). Early life exposure to 1959–1961 Chinese famine exacerbates association between diabetes and cardiovascular disease. J. Diabetes.

[20] Weisz G.M., Albury W.R. (2014). Hunger Whilst “In Utero” Programming Adult Osteoporosis. Rambam Maimonides Med. J..

[21] Zong L., Cai L., Liang J., Lin W., Yao J., Huang H., Tang K., Chen L., Li L., Lin L. (2019). Exposure to Famine in Early Life and the Risk of Osteoporosis in Adulthood: A Prospective Study. Endocr. Pract..

[22] Kin C.F.W., Shan W.S.Y., Shun L.J.C., Chung L.P., Jean W. (2007). Experience of famine and bone health in post-menopausal women. Int. J. Epidemiol..

[23] VanEvery H., Yang W.-H., Olsen N., Zhang X., Shu R., Lu B., Wu S., Cui L., Gao X. (2021). In Utero and Early Life Exposure to the Great Chinese Famine and Risk of Rheumatoid Arthritis in Adulthood. Arthritis Rheumatol..

[24] Liu C., Meng X., Zhang H., Yang F., Pan X., Tang K. (2021). Early-life famine exposure and rheumatoid arthritis in Chinese adult populations: —A Retrospective Cohort Study. BMJ Open.

[25] Xue A.-L., Wu S.-Y., Jiang L., Feng A.-M., Guo H.-F., Zhao P. (2017). Bone fracture risk in patients with rheumatoid arthritis. Medicine.

[26] Popkin B.M., Du S., Zhai F., Zhang B. (2010). Cohort Profile: The China Health and Nutrition Survey—Monitoring and understanding socio-economic and health change in China, 1989-2011. Int. J. Epidemiol..

[27] Popkin B.M., Paeratakul S., Ge K., Zhai F. (1995). Body weight patterns among the Chinese: Results from the 1989 and 1991 China Health and Nutrition Surveys. Am. J. Public Health.

[28] Zhang B., Zhai F.Y., Du S.F., Popkin B.M. (2014). The China Health and Nutrition Survey, 1989–2011. Obes. Rev..

[29] Zhai F.Y., Du S.F., Wang Z.H., Zhang J.G., Du W.W., Popkin B.M. (2014). Dynamics of the Chinese diet and the role of urbanicity, 1991–2011. Obes. Rev..

[30] Melaku Y.A., Gill T.K., Appleton S.L., Taylor A.W., Adams R., Shi Z. (2017). Prospective Associations of Dietary and Nutrient Patterns with Fracture Risk: A 20-Year Follow-Up Study. Nutrients.

[31] Jiang H., Yu Y., Li L., Xu W. (2021). Exposure to the Great Famine in Early Life and the Risk of Obesity in Adulthood: A Report Based on the China Health and Nutrition Survey. Nutrients.

[32] Wang Z., Zou Z., Wang S., Yang Z., Ma J. (2019). Chinese famine exposure in infancy and metabolic syndrome in adulthood: —Results from the China Health and Retirement Longitudinal Study. Eur. J. Clin. Nutr..

[33] Li C., Lumey L.H. (2017). Exposure to the Chinese famine of 1959–61 in early life and long-term health conditions: A Systematic Review and Meta-Analysis. Int. J. Epidemiol..

[34] Shi Z., Taylor A.W., Riley M., Byles J., Liu J., Noakes M. (2018). Association between dietary patterns, cadmium intake and chronic kidney disease among adults. Clin. Nutr..

[35] Ainsworth B.E., Haskell W.L., Whitt M.C., Irwin M.L., Swartz A.M., Strath S.J., O’Brien W.L., Bassett D.R., Schmitz K.H., Emplaincourt P.O. (2000). Compendium of Physical Activities: An Update of Activity Codes and MET Intensities. Med. Sci. Sports Exerc..

[36] Marcus E.-L., Menczel J. (2009). Experience of famine and bone health in post-menopausal women. Int. J. Epidemiol..

[37] Tarry-Adkins J.L., Ozanne S.E. (2017). Nutrition in early life and age-associated diseases. Ageing Res. Rev..

[38] Zheng J., Feng Q., Zheng S., Xiao X. (2018). Maternal nutrition and the developmental origins of osteoporosis in offspring: Potential mechanisms and clinical implications. Exp. Biol. Med..

[39] Cooper C., Fall C., Egger P., Hobbs R., Eastell R., Barker D. (1997). Growth in infancy and bone mass in later life. Ann. Rheum. Dis..

[40] Javaid M.K., Crozier S.R., Harvey N.C., Gale C.R., Dennison E.M., Boucher B.J., Arden N.K., Godfrey K.M., Cooper C. (2006). Maternal vitamin D status during pregnancy and childhood bone mass at age 9 years: A Longitudinal Study. Lancet.

[41] Pettifor J.M., Moodley G.P. (1997). Appendicular Bone Mass in Children with a High Prevalence of Low Dietary Calcium Intakes. J. Bone Miner. Res..

[42] Subramanian S., Huq S., Yatsunenko T., Haque R., Mahfuz M., Alam M.A., Benezra A., DeStefano J., Meier M.F., Muegge B.D. (2014). Persistent gut microbiota immaturity in malnourished Bangladeshi children. Nature.

[43] Shi Z. (2019). Gut Microbiota: An Important Link between Western Diet and Chronic Diseases. Nutrients.

[44] Das M., Cronin O., Keohane D.M., Cormac E.M., Nugent H., Nugent M., Molloy C., O’toole P.W., Shanahan F., Molloy M.G. (2019). Gut microbiota alterations associated with reduced bone mineral density in older adults. Rheumatology.

[45] Savitsky B., Manor O., Lawrence G., Friedlander Y., Siscovick D.S., Hochner H. (2021). Environmental mismatch and obesity in humans: The Jerusalem Perinatal Family Follow-Up Study. Int. J. Obes..

