# Supplementary material for: Exposure to Chinese Famine during Early Life Increases the Risk of Fracture during Adulthood
Source: Nutrients. 2022 Mar 3;14(5):1060. doi: 10.3390/nu14051060 (PMC8912709; doi:10.3390/nu14051060)
Supplement: Supplementary file 1 [file nutrients-14-01060-s001.zip › nutrients-1606432-supplementary.pdf]

Figure S1 Study sites of China Health and Nutrition Survey

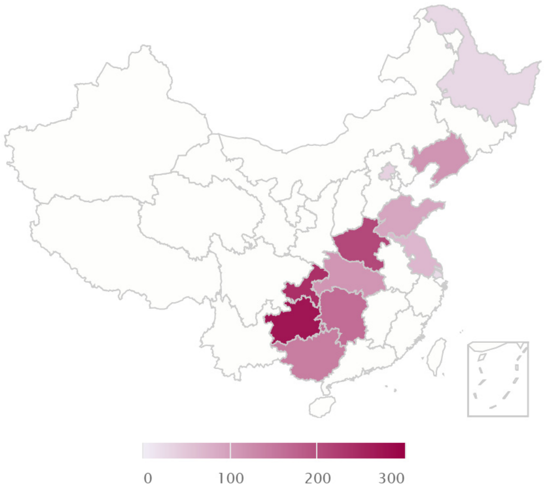

Values represents excessive mortality (%) during 1959-1961 famine.

Data source for excessive mortality: Luo, Z.; Mu, R.; Zhang, X. Famine and Overweight in China. *Review of Agricultural Economics* **2006**, *28*, 296-304

Figure S2 Incidence rate (per 1000) of fracture by study region among participants born between 1952 and 1964 who attended the China Health and Nutrition Survey between 1997 and 2015

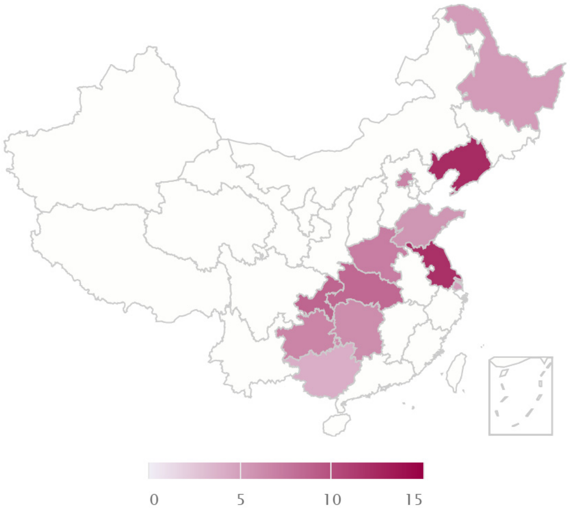

Table S1 Factor loadings of dietary patterns among participants born between 1952 and 1964 who attended the China Health and Nutrition Survey between 1997 and 2015

	Pattern 1 (Traditional south pattern)	Pattern 2 (Modern pattern)
Rice	**0.74**	**-0.24**
Wheat	**-0.69**	-0.14
Whole grain	**-0.42**	-0.03
Pork	**0.41**	0.27
Fish	**0.37**	**0.29**
Deep fried products	**-0.30**	**0.24**
Fresh vegetable	**0.27**	-0.10
Poultry	**0.26**	**0.25**
Dry tofu	**0.25**	0.11
Salted vegetable	**0.22**	-0.09
Offal	**0.20**	0.09
Tubers	-0.17	-0.16
Bean thread noodle	-0.08	0.03
Wine	0.02	-0.01
Fruit	-0.03	**0.45**
Milk	-0.06	**0.42**
Fast food	-0.20	**0.41**
Eggs	-0.16	**0.36**
Soy milk	-0.20	**0.35**
Shrimp	0.10	**0.35**
Fungus	0.07	**0.32**
Cake	-0.15	**0.28**
Yoghurt	-0.04	**0.25**
Nuts	0.04	**0.23**
Beer	0.08	**0.23**
Beef	0.17	**0.22**
Lamb	-0.02	**0.21**
Others	0.03	**0.21**
Beverage	-0.01	0.16
Sugar	0.00	0.15
Spirit	0.06	0.15
Legume	0.03	-0.09
Fresh bean	-0.01	-0.09
Tofu	-0.03	-0.04
Milk powder	0.02	0.04
Variance explained (%)	6.0	5.5
